# A method for freeze-fracture and scanning electron microscopy of isolated mitochondria

**DOI:** 10.1016/j.mex.2018.05.006

**Published:** 2018-05-19

**Authors:** Julie A. MacDonald, William H. Fowle, Ellie Shin, Dori C. Woods

**Affiliations:** Department of Biology, Northeastern University, Boston, MA, 02115, United States

**Keywords:** Freeze-fracture scanning electron microscopy of isolated mitochondria, Organelle, Ultrastructure, Protocol, Mitochondria, Scanning electron microscopy

## Abstract

Electron microscopy as a methodology for the study of mitochondria based on morphological features is a standard technique that has experienced little evolution over the course of several decades. This technology has identified heterogeneity of mitochondria populations across both whole tissues, as well between individual cells, using primarily ultrathin sections for transmission electron microscopy (TEM). However, this technique constrains the evaluation of a sample to a single two-dimensional plane. To overcome this limitation, scanning electron microscopy (SEM) has been successfully utilized to observe three-dimensional mitochondria structures within the complex microenvironment containing total cellular components. In response to these dual technical caveats of existing electron microscopy protocols, we developed a methodology to evaluate the three-dimensional ultrastructure of isolated mitochondria, utilizing a freeze-fracture step and rigorous preservation of sample morphology. This protocol allows for a more high-throughput analysis of mitochondria populations from a specimen of interest, as the sample has been previously purified, as well as a finer resolution of complex intra-mitochondrial structures, using the depth of field created by SEM.

• Protocol designed for SEM of isolated mitochondria samples.
• SEM visualizes mitochondria ultrastructure in 3-D.
• Freeze-fracture creates cross-sectional plane for view of interior organelle structures.

## Graphical abstract

**Figure:**
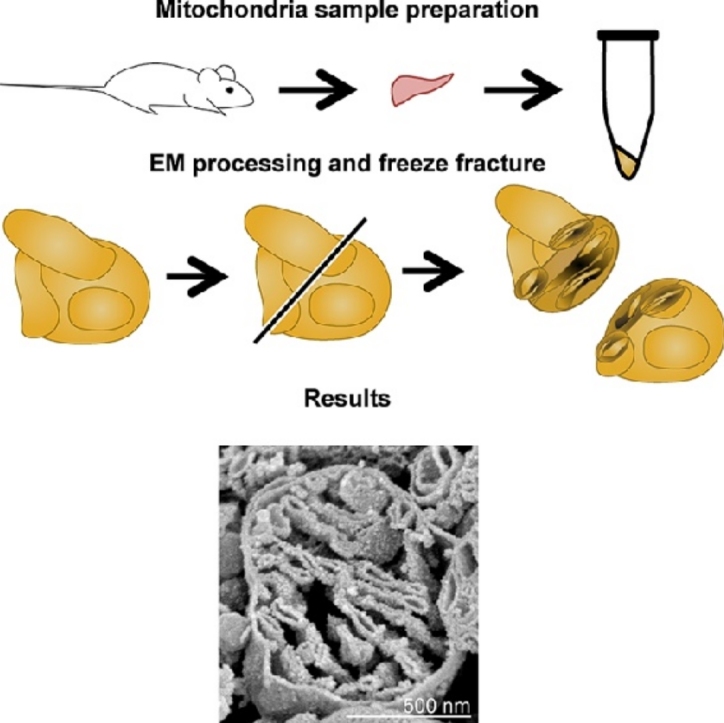

Specifications Table [please fill in right-hand column of the table below]

**Table:** 

Subject area	• *Biochemistry, Genetics and Molecular Biology*
More specific subject area	*Electron Microscopy; Mitochondria Biology*
Method name	*Freeze-fracture scanning electron microscopy of isolated mitochondria*
Name and reference of original method	*MacDonald, Julie A., William H. Fowle, and Dori C. Woods. "New insights on mitochondrial heterogeneity observed in prepared mitochondrial samples following a method for freeze-fracture and scanning electron microscopy." Micron.*[1]
Resource availability	*N/A*

## Method details

For the methodology provided here, mitochondria were isolated from cryopreserved C57BL/6 mouse tissue, however the method is applicable for many specimens of interest, including cultured cell lines or primary tissue samples from patient biopsy, for two examples—simply adjust the mitochondria isolation step as desired (*i.e.* use Mitochondria isolation kit (Product # 89,874; Pierce, Rockford, IL, USA) for cultured cells). For any experiments using research animals, ensure all protocols are reviewed and approved by the appropriate institutional animal care and use committee.

## Materials

Specimen processing:

• C57BL/6 mice (Charles River Laboratories, Wilmington, MA, USA)
• Mitochondria isolation kit (Product # 89801; Pierce, Rockford, IL, USA)

Reagents:

•
  ○ Carbon Dioxide (CO_2_; Product # CD 50; Airgas, Radnor, PA, USA)
  ○ Dimethyl sulfoxide (DMSO; Product # D2650;Sigma, St. Louis, MO, USA)
  ○ D-mannitol (Product # 125345000; Thermo Fisher Scientific, Waltham, MA, USA)
  ○ Ethanol (Product # BP2818500; Thermo Fisher Scientific, Waltham, MA, USA)
  ○ Fetal Bovine Serum (FBS; Product # PS500 A; Peak Serum, Wellington, CO, USA)
  ○ Formaldehyde (Product # 15700; Electron Microscopy Sciences, Hatfield, PA, USA)
  ○ Glutaraldehyde (Product # 16000; Electron Microscopy Sciences, Hatfield, PA, USA)
  ○ Nitrogen (LN_2_; Product # NI 240LT22; Airgas, Radnor, PA, USA)
  ○ Osmium tetroxide (Product # 19150; Electron Microscopy Sciences, Hatfield, PA, USA)
  ○ Phosphate buffered saline (PBS; Product # 20012027; Thermo Fisher Scientific, Waltham, MA, USA)
  ○ Potassium Chloride (KCl; Product # BP366-500; Thermo Fisher Scientific, Waltham, MA, USA)
  ○ Potassium phosphate monobasic (KH_2_PO_4_; P X 1565-1; Millipore Sigma, Burlington, MA, USA)
  ○ Sodium Cacodylate (Product # 21135; Electron Microscopy Sciences, Hatfield, PA, USA)
  ○ Sucrose (Product # BP220-1; Thermo Fisher Scientific, Waltham, MA, USA
  ○ Tannic Acid (Product # 21700; Electron Microscopy Sciences, Hatfield, PA, USA)
  ○ Tris−HCl (Product # T1068; Teknova, Hollister, CA, USA)
  ○ Water (Ultrapure; Product # SH30538.01; GE Healthcare Life Sciences, Logan, UT, USA)

Equipment:

• 1.5 mL microcentrifuge tubes, polypropylene (Product # 5,408,133; Thermo Fisher Scientific, Waltham, MA, USA)
• 19 x 51 mm glass vial (Product # 0333926C; Thermo Fisher Scientific, Waltham, MA, USA)
• Aluminum dissecting pan (Product # 1,381,460; Thermo Fisher Scientific, Waltham, MA, USA)
• Aluminum mount (Product # 75,100; Electron Microscopy Sciences, Hatfield, PA, USA)
• Aluminum weighing spatula (Product # 14,371 A; Thermo Fisher Scientific, Waltham, MA, USA)
• Carbon adhesive tabs (Product # 77,816; Electron Microscopy Sciences, Hatfield, PA, USA)
• Colloidal graphite (Product # 12,650; Electron Microscopy Sciences, Hatfield, PA, USA)
• Critical point dryer (Samdri^®^-PVT-3D; Tousimis, Rockland, MD, USA)
• Forceps (Product # **78310-0**; Electron Microscopy Sciences, Hatfield, PA, USA)
• High-resolution sputter coater (208HR (platinum); Cressington Scientific Instruments, Watford, UK)
• Microcentrifuge (Sorvall Legend Micro 21R; Thermo Fisher Scientific, Waltham, MA, USA)
• Pasteur Pipets and bulb (Product # 1,367,820 A; Thermo Fisher Scientific, Waltham, MA, USA)
• Polyurethane Ice Bucket (Product # 0,259,145; Thermo Fisher Scientific, Waltham, MA, USA)
• Razor blades (Product # 12,640; Thermo Fisher Scientific, Waltham, MA, USA)
• Field emission scanning electron microscope (Hitachi S-4800; Hitachi Medical Corporation, Tokyo, Japan)

Protocol

Isolation of mitochondria:

1 Euthanize C57BL/6 mice and dissect liver tissue (or tissue of interest).
  a Tissues can be used immediately for processing, or
  b Tissues can be cryopreserved in freezing medium containing fetal bovine serum (FBS) supplemented with 10% dimethyl sulfoxide (DMSO), slow frozen, and stored at −80 °C until time of use.
2 Dissociate isolated tissue into small (∼200 mg) samples to be used for mitochondria isolation, as per the manufacturers protocol, in this case: Mitochondria isolation kit (Product # 89,801; Pierce, Rockford, IL, USA)
  a Many kits are commercially available for mitochondrial isolation, as well as published protocols.
3 Collect isolated mitochondria from Step 2 in 1.5 mL microcentrifuge tubes, and wash once by resuspension in 1 mL of a chilled (4 °C) respiration-compatible buffer.
  a Our preferred buffer contains 225 mM D-mannitol, 75 mM sucrose 10 mM KCl, 10 mM Tris−HCl, 5 mM KH_2_PO_4_ at pH 7.0.
4 Pellet washed mitochondria from Step 3 were by centrifugation at 12,000xg for five minutes, and carefully removed by pipet aspiration, and
5 Fix pellet in a primary fixative of 2.5% glutaraldehyde and 2.5% formaldehyde in 0.1 M sodium cacodylate buffer, pH 7.2 overnight (16 h) at 4 °C.
  a Use appropriate personal protective equipment when working with fixatives. Many steps should be completed in a chemical fume hood.

Processing for electron microscopy:

1 Using forceps, carefully remove fixed mitochondrial pellet from “Isolation of mitochondria, Step 5″ and place in a borosilicate glass vial containing 3 mL wash buffer. Incubate at room temperature (22 °C) for 10 min. Repeat for two additional washes.
  a Wash buffer: 0.1 M sodium cacodylate buffer, pH 7.2
2 Remove final wash buffer by pipet aspiration, and post-fix in 1 mL 1% osmium tetroxide in 0.1 M sodium cacodylate buffer, pH 7.2 for two hours at room temperature (22 °C), protected from light.
3 Remove post-fixation buffer and wash pellets a total of three times with 3 mL wash buffer for 10 min at room temperature (22 °C).
4 Impregnate samples with DMSO by removing final wash buffer and incubating samples with a 25% solution of DMSO in 0.1 M sodium cacodylate buffer, pH 7.2 for 30 min at room temperature (22 °C).
  a During the first DMSO incubation, prepare a suitable workstation for the LN2 freeze fracture. For this study, we used a small aluminum instrument tray fitted over a polyurethane ice bucket, validated for use with liquid nitrogen, as well as stainless steel forceps and razor blades, and an aluminum weighing spatula to use as a hammer.
5 Remove first DMSO solution and replace with a 50% solution of DMSO in 0.1 M sodium cacodylate buffer, pH 7.2 and incubate samples for 30 min at room temperature (22 °C).
  a During the second DMSO incubation, cool all instruments in a bath of liquid nitrogen, and allow temperature to stabilize for at least 5 min prior to use. Use appropriate personal protective equipment when working with liquid nitrogen.

**Fig. 1:**
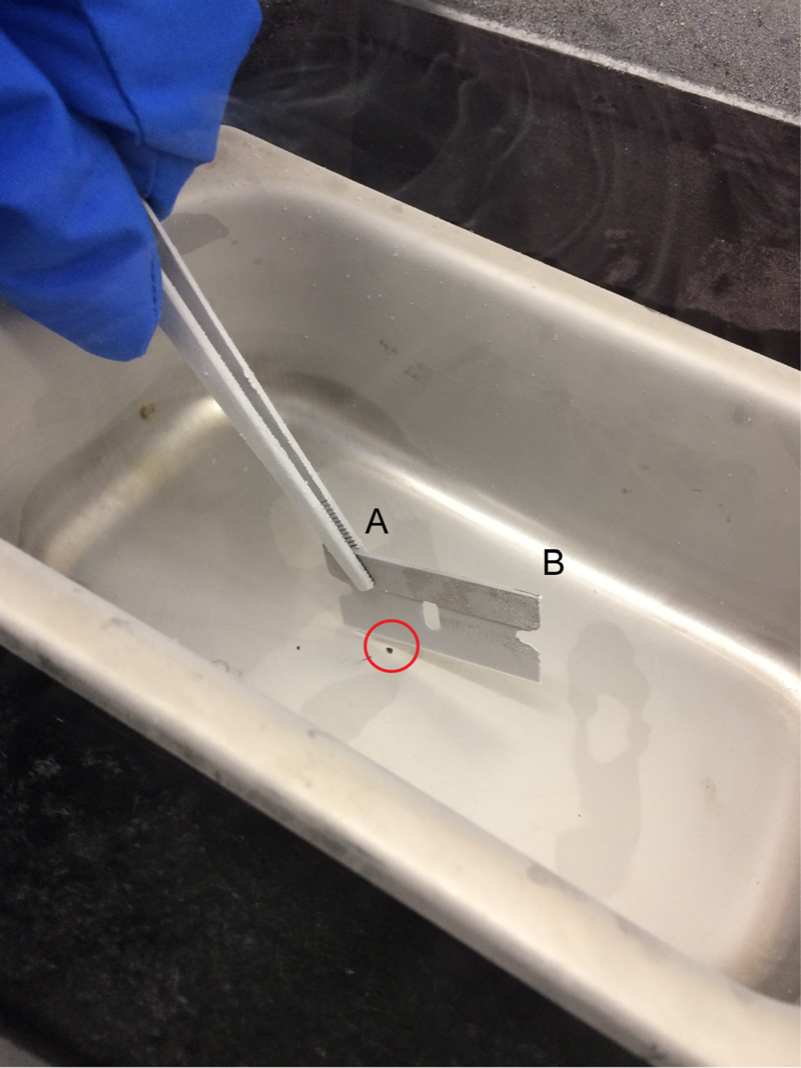
Setup for freeze-fracture of isolated mitochondria samples. Example image for processing of Step 2 of “Processing for electron microscopy.” Note the sample, indicated by the red circle, positioned on a solid metal surface prechilled with liquid nitrogen. The razor blade is held securely in place by forceps above the sample (A), making direct contact with fracture surface. Elevating the razor blade at a 45° angle over the sample creates an elevated target corner (B) to be struck firmly in a downward motion to cleanly fracture the pellet.
3 Carefully and rapidly collect fractured pellet shards and thaw in a fresh solution containing 50% DMSO in 0.1 M sodium cacodylate buffer, pH 7.2 and incubate samples for 30 min at room temperature (22 °C) to gently thaw.
  a Limit manipulation of the samples to reduce risk of damage to fractured surfaces.
4 Repeat “Processing for electron microscopy” Steps 1–3.
5 Remove final wash buffer by micropipette aspiration, and macerate samples in 3 mL of 0.1% osmium tetroxide in 0.1 M sodium cacodylate buffer, pH 7.2 buffer at room temperature (22 °C) overnight (16 h).
6 Remove maceration buffer and wash, as described in “Processing for electron microscopy, Step 1.″
7 Remove final wash buffer and incubate samples for one hour in a 1% tannic acid solution in 0.1 M sodium cacodylate buffer, pH 7.2 at room temperature (22 °C).
8 Remove tannic acid solution, and wash as described in “Processing for electron microscopy, Step 1.″
9 Remove final wash buffer incubate samples in a final post fixation solution containing 1% osmium tetroxide in 0.1 M sodium cacodylate buffer for one hour at room temperature (22 °C), protected from light.
10 Remove post fixation solution wash as described in “Processing for electron microscopy, Step 1.″
11 Remove final wash buffer and dehydrate samples by moving mitochondria fragments through a graded series of ethanol washes, each wash for 10 min at room temperature.
  a Ethanol dehydration steps: 30%, 50%, 70%, 85%, 95%, and 100%, using molecular grade ethanol diluted in ultrapure water.
12 Proceed to critical point drying of samples, as per standard processing for SEM.
  a For example, in brief:
    i Cover sample in 100% ethanol, transfer into Critical Point Dryer, such as Samdri®-PVT-3D (Tousimis, Rockland, MD, USA).
    ii Cool the chamber to −10 °C
    iii Fill the chamber with liquid CO2, close system, and incubate for 2 min.
    iv Purge chamber, and recool, repeating steps ii-iv for 9–10 cycles.
    v Fill the chamber with liquid CO2 and begin heating chamber to critical point.
    vi Incubate at critical point for 4 min.
    vii Begin to bleed the chamber and slowly reduce pressure until sample can be retrieved.
13 Once dried, mount sample fragments onto a one-inch aluminum sample mount using double-sided carbon adhesive tabs and a colloidal graphite solution, as needed, and allowed to air dry.
  a When mounting, careful attention should be paid to ensure fractured surfaces are readily available for evaluation by SEM.
14 Coat mounted samples using a high-resolution 4 nm sputter coating of platinum, and analyzed samples using a field emission scanning electron microscope such as the Hitachi S-4800 (Hitachi Medical Corporation, Tokyo, Japan).
15 See Fig. 2 for example images.

Freeze fracture and sample mounting:

1 Using forceps, carefully remove DMSO impregnated samples from “Processing for electron microscopy, Step 5″ and place on prepared frozen surface.
  a Watch for freezing of residual DMSO surrounding sample to anchor sample to metal surface.
2 Firmly grasp one corner of a razor blade with forceps and place at 45° angle over pellet, making contact with the metal surface with one corner of the blade edge. Using a hammer, strike the elevated corner of the razor blade (not supported by the forceps) in a downward motion to fracture the pellet (Fig. 1)
  a Repeat as needed until desired number of fragments is obtained. Fracture should create a planar surface through the mitochondrial pellet.

**Fig. 2:**
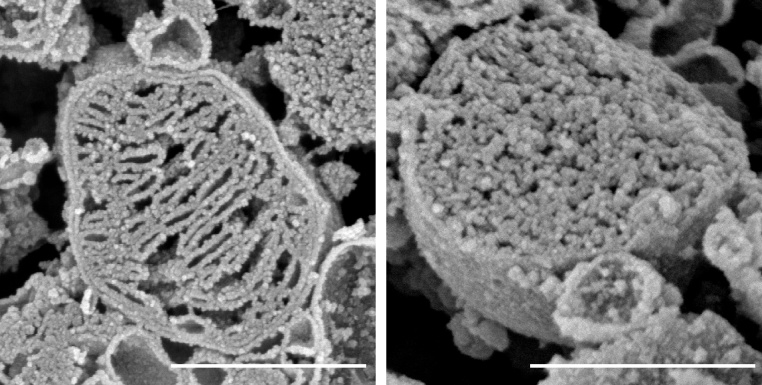
Example images resulting from method for freeze-fracture and scanning electron microscopy of isolated mitochondria. Two representative images demonstrating capability to finely resolve mitochondria ultrastructure, including outer membrane, inner membrane, and cristae structures. Scale bars indicate 500 nm.
