# Supplementary material for: A method for freeze-fracture and scanning electron microscopy of isolated mitochondria
Source: MethodsX. 2018 May 19;5:593–8. doi: 10.1016/j.mex.2018.05.006 (PMC6031756; doi:10.1016/j.mex.2018.05.006)
Supplement: Supplementary file 1 [file mmc1.docx]

**Supplementary material *and/or* Additional information:**

This work represents an extension of several influential studies in scanning electron microscopy [2–4] to focus on the ability to resolve isolated mitochondria, however these resources could also be adapted for use in other subcellular structures. We would like to emphasize the requirement of physical manipulation of the sample (*e.g.* Moving the sample through each solution and completing the fracture step) and would strongly recommend careful consideration of sample input to avoid impractically small samples for processing. One recommendation would be to embed smaller samples in a carrier substrate, such as an agarose block, to ease strain of processing. Additionally, fractured fragments that may be too small to continue processing for evaluation by SEM have been successfully embedded and sectioned for evaluation for transmission electron microscopy.

The suggested timing of the procedure steps reflects what we found to be most practical, in our hands. However, it may be possible for the user to reduce post-fix and maceration times to one hour to accommodate processing within a single day. We were most satisfied with results pausing sample processing in an overnight maceration incubation. Samples may also be stored overnight following dehydration to a solution containing 70% ethanol. As with any procedure this protocol should be optimized for sample type and equipment available.

References

[2] K. Tanaka, Scanning electron microscopy of intracellular structures, 1980. doi:10.1517/17425247.2015.974547.

[3] K. Tanaka, A. Mitsushima, A preparation method for observing intracellular structures by scanning electron microscopy, J. Microsc. 133 (1984) 213 – 222.

[4] T. Murakami, A Revised Tannin - Osmium Method for Non - Coated Scan ning Electron Microscope Specimens, Arch. Histol. Jpn. 36 (1974) 189 – 193
